# Properties, Extraction Methods, and Delivery Systems for Curcumin as a Natural Source of Beneficial Health Effects

**DOI:** 10.3390/medicina56070336

**Published:** 2020-07-03

**Authors:** Aleksandra Zielińska, Henrique Alves, Vânia Marques, Alessandra Durazzo, Massimo Lucarini, Thais F. Alves, Margreet Morsink, Niels Willemen, Piotr Eder, Marco V. Chaud, Patricia Severino, Antonello Santini, Eliana B. Souto

**Affiliations:** 1 Department of Pharmaceutical Technology, Faculty of Pharmacy, University of Coimbra, Pólo das Ciências da Saúde, Azinhaga de Santa Comba, 3000-548 Coimbra, Portugal; zielinska-aleksandra@wp.pl (A.Z.); henrique.pinho.alves@gmail.com (H.A.); vansmarq12@gmail.com (V.M.); 2 Polish Academy of Sciences, Institute of Human Genetics, Strzeszyńska 32, 60-479 Poznań, Poland; 3 CREA-Research Centre for Food and Nutrition, Via Ardeatina 546, 00178 Rome, Italy; alessandra.durazzo@crea.gov.it (A.D.); massimo.lucarini@crea.gov.it (M.L.); 4 Laboratory of Biomaterials and Nanotechnology, University of Sorocaba-UNISO, Sorocaba, São Paulo 18023-000, Brazil; thaisfrancinealves1@gmail.com (T.F.A.); marco.chaud@prof.uniso.br (M.V.C.); 5 Center for Biomedical Engineering, Department of Medicine, Brigham and Women& Hospital, Harvard Medical School, 65 Landsdowne Street, Cambridge, MA 02139, USA; m.a.j.morsink@student.utwente.nl (M.M.); n.g.a.willemen@student.utwente.nl (N.W.); pattypharma@gmail.com (P.S.); 6 Translational Liver Research, Department of Medical Cell BioPhysics, Technical Medical Centre, Faculty of Science and Technology, University of Twente, 7522 NB Enschede, The Netherlands; 7 Department of Developmental BioEngineering, Faculty of Science and Technology, Technical Medical Centre, University of Twente, 7522 NB Enschede, The Netherlands; 8 Department of Gastroenterology, Dietetics and Internal Diseases, Poznan University of Medical Sciences, Przybyszewskiego 49, 60-355 Poznań, Poland; piotr.eder@op.pl; 9 Nanomedicine and Nanotechnology Laboratory (LNMed), Biotechnological Postgraduate Program, and Institute of Technology and Research (ITP), University of Tiradentes (Unit), Av. Murilo Dantas 300, Aracaju 49010-390, Brazil; 10 Tiradentes Institute, 150 Mt Vernon St, Dorchester, MA 02125, USA; 11 Department of Pharmacy, University of Napoli Federico II, Via D. Montesano 49, 80131 Napoli, Italy; 12 CEB—Centre of Biological Engineering, University of Minho, Campus de Gualtar, 4710-057 Braga, Portugal

**Keywords:** curcumin, nanopharmaceutics, nanoparticles, cancer, health properties

## Abstract

This review discusses the impact of curcumin—an aromatic phytoextract from the turmeric (*Curcuma longa*) rhizome—as an effective therapeutic agent. Despite all of the beneficial health properties ensured by curcumin application, its pharmacological efficacy is compromised in vivo due to poor aqueous solubility, high metabolism, and rapid excretion that may result in poor systemic bioavailability. To overcome these problems, novel nanosystems have been proposed to enhance its bioavailability and bioactivity by reducing the particle size, the modification of surfaces, and the encapsulation efficiency of curcumin with different nanocarriers. The solutions based on nanotechnology can improve the perspective for medical patients with serious illnesses. In this review, we discuss commonly used curcumin-loaded bio-based nanoparticles that should be implemented for overcoming the innate constraints of this natural ingredient. Furthermore, the associated challenges regarding the potential applications in combination therapies are discussed as well.

## 1. Introduction

**Figure 1:**
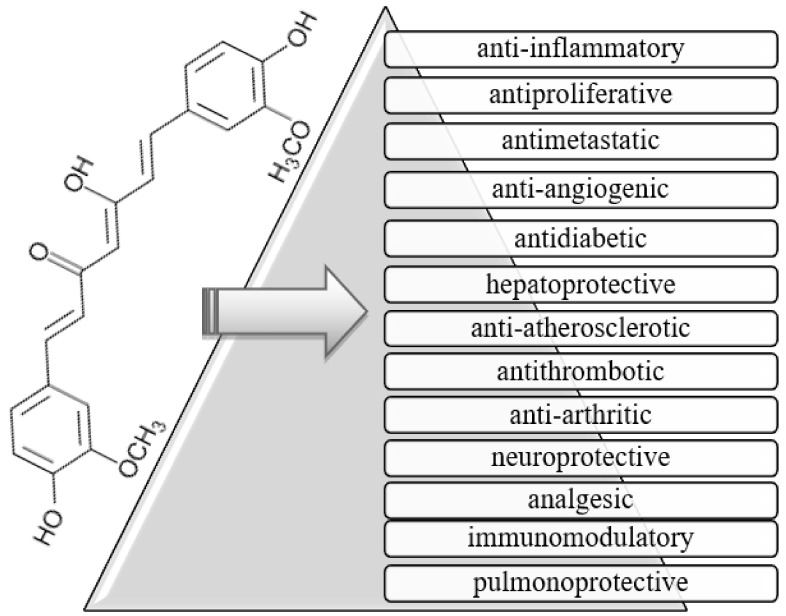
Chemical structure and selected properties of curcumin (C_21_H_20_O_6_).

Curcumin, a curcuminoid [1], is a bioactive component representing 1.5–3 wt.% [2] of the rhizome of turmeric (*Curcuma longa*) [3], also known as diferuloylmethane. This yellow pigmented powder can be obtained by grinding the rhizome of turmeric [4], owing the color to the curcumin present [1,5]. It has been used over the centuries for cooking, especially in Asia, as well as for treatment due to its curing properties in diseases, such as dysentery, chest congestion, and pain. Moreover, it has been known as a strong antioxidant [3]. Over the past half a century, curcumin has received growing interest in biological [6,7,8,9,10,11,12,13,14], pharmacological [15,16,17,18,19], and nutraceutical research [20,21,22,23,24,25,26]. Interestingly, it was in 1949 that its antibacterial properties were discovered [1]. Since then, many studies have proved that curcumin also possesses other potential beneficial properties, such as anti-inflammatory [27,28], antiproliferative [29,30], antimetastatic [31,32], anti-angiogenic [29,33], antidiabetic [34,35,36], hepatoprotective [37,38,39], anti-atherosclerotic [40,41], antithrombotic [42], wound healing [43], anti-cancer [44], anti-arthritic [45], neuroprotective [46,47], analgesic [45], immunomodulatory [48,49,50,51], and pulmonoprotective [52,53] properties, among many other effects [1,34,54,55], summarized in Figure 1.

Curcumin (C_21_H_20_O_6_), as shown in Figure 1, also named [1,7-bis (4-hydroxy-3- methoxyphenyl)-1,6-heptadiene-3,5-dione] [39], has a molecular weight of 368.38 g/mol and it is solid at room temperature. It is a hydrophobic molecule, mostly insoluble in water (only 30 nM can be dissolved) and poorly soluble in hydrocarbon solvents [1]; however, curcumin is very soluble in polar solvents [3]. The chemical structure of curcumin contains two aromatic ring systems with o-methoxy phenolic groups [3]. Thanks to three functional groups—an aromatic o-methoxy phenolic group, α,β-unsaturated β-diketo moiety, and a seven carbon chain—the component is biologically active [1].

Despite all its benefits, curcumin has low bioavailability [1,56,57]. Because an oral dose of curcumin has a reduced absorption, the molecule is rapidly metabolized in the intestine and the liver [54] by aldo-keto reductase and subsequently eliminated rapidly [34], limiting those benefits. Its metabolization results in different curcumin conjugates that have either active or inactive form. These, in turn, are further transformed into their extractable forms, glucuronide and sulfate conjugates. These compounds can bond with curcumin through its exposed -OH and -OCH_3_ sites to form conjugates, whose bonds can be broken by glucuronidases and sulfatases, respectively [54].

The main metabolic pathways of curcumin are conjugation and reduction. When orally administered, curcumin undergoes conjugation resulting in the formation of glucuronide and curcumin sulfates in the intestine and liver. If administered by intraperitoneal or other systemic routes, curcumin is transformed into hexahydrocurcumin and octahidrocurcumin by reduction. The latter are converted into monoglucuronic conjugates (the main metabolites), or curcumin glucuronide, dihydrocurcumin glucuronide, and tetrahydrocurcumin glucuronide (these being less active) [58]. Additionally, curcumin can degrade in a quick manner into other substances when present in physiological conditions. These include ferulic acid, ferulic aldehyde, feruloyl methane, and vanillin, among others [3].

Concerning the benefits of curcumin, it is known to be capable of modulating growth factors, enzymes, transcription factors, kinase, inflammatory cytokines, and proapoptotic and antiapoptotic proteins, being a versatile molecule to treat several diseases. Specific in wound healing, the curcumin acts in the inflammatory phase, reducing the cytokines as tumor necrosis factor (TNF-α), interleukin-1 (IL-1), and inhibits the nuclear factor-kB (NK-B). A similar activity is observed in cancer treatment. Curcumin reduces the inflammatory condition using different pathways. It interacts with immune mediators and shows antioxidant activity [43,44]. Due to its easy metabolization, studies have been conducted to enhance the stability and bioavailability of curcumin through molecular modification [1,3]. In the last 20 years, a vast number of drug delivery systems, such as micelles, liposomal vesicles, nanoemulsions, phospholipid complexes, and polymeric implants, has been developed [54], enabling their use for therapeutic prevention or risk reduction in the precancer stage or even across the blood–brain barrier, allowing for the treatment of neurodegenerative diseases [1].

## 2. Methods for Curcumin Extraction

Curcuminoids and oleoresin from *Curcuma longa* are susceptible to degradation by oxidation catalyzed by light and heat. Currently, the extraction methods of curcuminoid and oleoresin have favored techniques in which heating can be easily managed. After the extraction of curcuminoids, pigments and other chemically stable compounds are extracted by percolation (boiler and reflux), steam-, and hydro-distillation. Table 1 shows the different extraction techniques that have been used for the extraction of curcuminoids, oleoresin, pigments, and other compounds from *Curcuma* longa for use in pharmaceutical and nutraceutical products.

Carbon dioxide as a supercritical antisolvent (SAS/CO_2_-FSC) has been used to extract oleoresin and curcuminoids from *Curcuma longa* and *Curcuma amada*. Carbon dioxide is inert, readily available, non-toxic, non-corrosive, non-flammable, recyclable, and it is stable at mild conditions (i.e., 37 °C and 72.8 bar), which enables it to extract heat-labile compounds and also preserve their quality [59]. Nagavekar and Singhal improved the extraction efficiency upon the use of 30% ethanol as a modifier or with enzymatic before treatment with CO_2_-FSC [60].

Deep eutectic solvent (DES) is a micro-extraction method with selective fast, environmentally friendly and good thermal stability. It is a system formed from a eutectic mixture which can contain a variety of ionized species with select properties of the ionic solvents. Generally, the vortex is used to assist the extraction from edible essential oil samples [61].

An aqueous two-phase extraction system, combined with a dispersive liquid–liquid microextraction method using imidazolium as an ultrasound-assisted ionic liquid, has been used for the separation of curcuminoids. In this method, when the ionic solution is acidified (pH < 4.0), the recovery rate is 93% [62].

Supramolecular solvents attract the intentions of scientist in the field of the extraction of organic compounds. Using ultrasonic-assisted restricted access supramolecular solvents is an emerging trend for the separation of phenolic bioactive compounds. The specific properties of supramolecular solvents are considered for liquid-phase extraction due to their physical properties. The amount of extraction solution using supramolecular solvents can be reduced, and thus the excessive discharge of organic solvents into the waste can be significantly decreased [63,64]. Menghwar et al. [65] employ ultrasonic-assisted restricted access supramolecular solvent-based microextraction (UA-RAS-LPME) using long chain alkanols or acids (C7–C14) to extract curcumin. Here, the -OH and -O functional groups of curcumin can form hydrogen bonds with the supermolecular solvents, and van der Waals and dispersion forces attract the apolar chain of curcumin. This creates a double way to extract curcumin to complete the procedure within 20 min using ultrasonic waves, providing an attractive way to extract the active compound.

Other extraction methods are based on the extraction of turmeric oil and oleoresin. Turmeric oil can offer bactericidal and fungistatistical activities. In order to extract turmeric oil, researchers have used steam distillation, hydro-distillation, and extraction using hexane. Hexane was combined with the oils after curcumin extraction and heated to 60 °C three times for one hour. The solvent was removed, which resulted in successful turmeric oil extraction [66]. Steam distillation is used with volatile solvents to extract turmeric essential oils. Here, an autoclave machine was used to create controlled pressure and controlled temperatures to pass through the turmeric, after which the steam was cooled down using water and the essential oils were obtained. Moreover, a similar technique was used with a volatile solvent, rather than the autoclave, where they were both heated to 40 °C. The extraction took place by separating the volatile solvent from the solid oil after cooling down and filtering [67]. Hydro-distillation is the last technique used to extract turmeric oil, where the turmeric oil is passed through a hydro-distillation machine, yielding the essential oils [66,67].

Conventional Soxhlet extraction is a traditional apparatus commonly used for the extraction of lipids and materials that are not water-soluble. Soxhlet can even store these substances, maintaining their properties. The study using the stock liquor after the isolation of curcumin from oleoresin contains approximately 40% oil. The isolation and identification of the antibacterial fractions from the leftover turmeric oleoresin were done by Soxhlet extraction. The best extraction was obtained with 5% ethyl acetate in hexane, while the ar-turmerone, turmerone, and curlone were identified as the significant compounds after gas chromatography analysis. The material showed antibacterial activity against *Bacillus cereus, Bacillus coagulans, Bacillus subtilis, Staphylococcus aureus, Escherichia coli,* and *Pseudomonas aeruginosa* [68].

The microwave-assisted extraction occurs in a way opposite to conventional heat extraction, rotation dipole, and ionic conduction. For microwave heating, it is necessary that the flask used for the extraction is made of a transparent material, such as quartz or Teflon. All of the material inside the bottle is heated directly and it is common to note that parts of the bottle containing the material to be analyzed remain at a temperature close to the environment immediately after being heated. Additionally, the material used in the system must be able to absorb the microwave energy and convert it into heat. Dried rhizomes were extracted by the microwave process and showed a reduction in the time of extraction and solvent use. Additionally, the rate of extraction was 27% more efficient than the conventional Soxhlet extraction, and the result can be attributed to the dual heating phenomenon of the solvent and sample matrix used [69].

**Table 1:** Methods, principles, and conditions for extraction of curcuminoids and oleoresin.

Methods	Conditions and Principles	Source of Extraction	References
Antisolvent supercritical solution (SAS)	Carbon dioxide Supercritical	Dried rhizomes collected from India and China	[59,60]
Vortex-assisted deep eutectic solvent (DES)	Emulsification liquid—liquid micro-extraction	Turmeric liquid extract obtained commercially	[61]
Liquid–liquid microextraction	Aqueous two-phase extraction using imidazolium and ultrasound	Mixture of curcuminoids obtained commercially	[62]
Ultrasound-assisted ionic liquid-dispersive	Liquid micro-extraction	Dried rhizomes obtained in Turkey market and power obtained commercially	[63,64]
Environment-responsive long chain acid (C7–C14)	Supramolecular solvents	Power obtained commercially	[65]
Microwave-assisted extraction	Microwave energy for analyte partition	Dried rhizomes from India	[69]
Soxhlet extraction	Percolation (boiler and reflux)	Mother liquor/curcumin oleoresin was collected from a local oleoresin industry	[68]
Steam-distillation	Fractional distillation based on boiling point	Dried rhizomes obtained in Brazil	[66]
Hydro-distillation	Vaporization–condensation cycle	Dried rhizomes obtained in Brazil	[67]

## 3. Curcumin-Loaded Colloidal Delivery Systems

**Figure 2:**
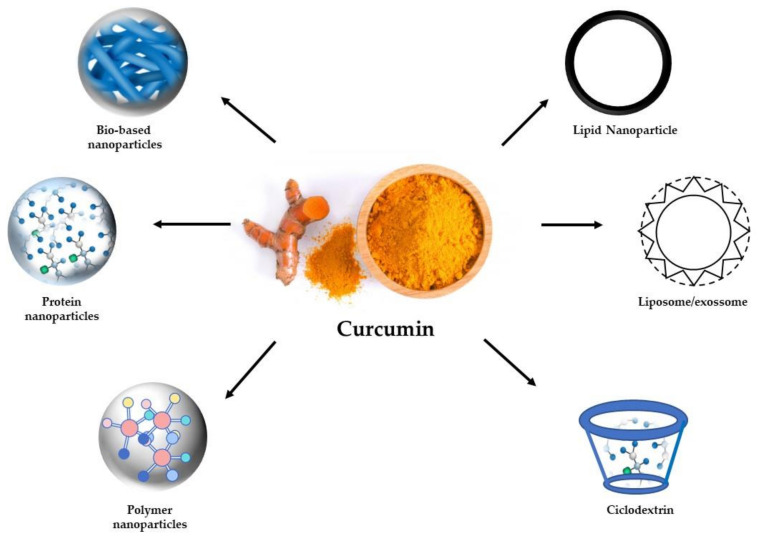
Different curcumin-loaded colloidal delivery systems.

In order to enhance the bioavailability and solubility of curcumin, nanoparticles (NPs), as a colloidal delivery systems, have been used for a few years [70,71] in line with the development of nanotechnologies [72,73] and emerging applications on the nutraceutical area [74,75,76,77,78,79,80,81,82,83]. There are diverse types of nanocarriers that have been used to deliver curcumin (synthetic and biological) for the treatment of advanced diseases, such as cancer and neurodegenerative disorders [84]. Their characteristics vary from one to another. This means that each one needs to be studied for its potential use. Nanocarriers have been used for curcumin delivery to circumvent the substance’s bioavailability, which greatly limits its use for therapeutic ends in patients [84,85]. Types include nanoparticles (NPs), liposomes, or ultrasound microbubbles [86,87]. Additionally, biopolymers can also be used as nanocarriers, which include chitosan, starch, zein, alginate, and silk, among others. Biopolymers have many advantages, including biocompatibility and biodegradability [88]. Exosomes—a biological nanocarrier secreted by cells—can also be used. It has been noted that the size of nanocarriers can alter the effect of their haul and may present some toxicity or immune response depending on the physical/chemical characteristics. The incorporation of curcumin into exosomes can vastly improve its solubility, stability, and, in general, bioavailability [89,90]. Consequently, high plasma concentrations can be acquired with lower dosages, reducing side effects and maintaining therapeutic efficacy [91]. Curcumin-loaded colloidal delivery systems are shown in Figure 2.

Another problem associated with curcumin is its time-consuming extraction [92]. However, a superior method using ultrasound has been discovered. This method not only reduces the extraction time but also it improves the yield of the process. As heat is not involved, there is no risk of heat-associated degradation [84].

### 3.1. Curcumin-Loaded Bio-Based Nanoparticles

Biopolymer nanoparticles (BpNPs) are colloidal structures that are assembled from one or more types of biopolymer molecules [91], which are biocompatible and biodegradable [84]. They allow us to improve drug stability and availability, by reducing its degradation, increasing absorption, and to target specific sites. Moreover, their sustained and controlled release system makes these particles very attractive. BpNPs are classified based on several different components, methods of preparation, and sizes [91]. The main type of BpNPs are the protein-based NPs, which include albumin, zein, and silk, further described below.

Albumin is the main protein in plasma, possessing high stability, non-immunogenicity, and biocompatibility. All of these features, coupled with the fact that it has a vast number of binding sites in its matrix, makes it a splendid nanocarrier, especially for curcumin, due to albumin’s high solubility compared to curcumin [93]. For example, human serum albumin (HSA) significantly improves the cellular intake of curcumin, and thereby increases its effects. In a study, HSA was used for curcumin carrying with the goal of having a redox-responsive deliverance, and the results were rather positive, showing an increased curcumin release in the presence of glutathione [91]. Another study related to bovine serum albumin (BSA) has shown, once again, promising results, such as pH and temperature resistance, while also showing an antioxidant effect [91,94].

Zein is a protein present in corn, mainly composed of non-polar amino acids, allowing for the encapsulation of hydrophobic compounds. According to a study, it has been proven that curcumin can be loaded in zein-based NPs [91]. The study has presented zein-hyaluronan NPs, and the results have shown high stability and a controlled release system in gastrointestinal simulation [95]. There were multiple studies carried out by using zein in combination with various reagents. Some of them concerned the use of zein with gelatin, alginate, and/or hyaluronan combinations [96], resulting in high stability and successful drug encapsulation. Whereas, zein NPs covered with quercetaget and hyaluronic acid resulted in high stability, decreased thermal degradation, and slow curcumin release [91,95].

Silk has several secondary structures, such as α-helix, β-sheets, and coil, among others, used for different types of functions. It becomes interesting due to its negative charge, high stability, and low toxicity [97]. Fibroin, as the main silk protein, has an array of properties that enable it to be an efficient biomaterial for drug delivery. For these reasons, several studies have shown promising results with the encapsulation of curcumin in silk fibroin NPs [98,99]. Curcumin-loaded silk fibroin NPs have proved higher efficacy in cytotoxicity against neuroblastoma cells than hepatocarcinoma cells [100], also displayed by the in vitro enhancement of antioxidant and anti-inflammatory properties and increase in healthy cell viability. After oral administration, the impact of different silk fibroin NP sizes affects their final properties, as silk fibroin NPs in the range of 800 nm have shown longer half-life and slower release, while smaller particles, around 200 nm, have presented a high maximum plasma concentration and higher bioavailability [91].

There are many other bio-based NPs. Among them there is rice bran albumin (RBA), which, combined with chitosan, can improve curcumin solubility and facilitate its encapsulation in NPs. These NPs have demonstrated cytotoxicity towards cancer cells [91]. Chitosan, a linear cationic heteropolymer, immediately allows cell permeability. This makes it a preferred nanocarrier, due to its antibacterial properties, low immunogenicity, and biocompatibility [91]. Based on keratin and Pluronic, which are thermo-responsive copolymers, hydrogel NPs have fabricated. Obtained nanoparticles have shown a size decrease with a temperature increase to 37 °C. A thermo-responsive release has also been observed. Polysaccharide NPs can also be used as nanocarriers due to their high stability, biocompatibility, and biodegradability. Alginate is a negative charged, pH-sensitive, and mucoadhesive soluble polysaccharide. Combined with chitosan, it is an efficient nanocarrier for curcumin, improving its stability, constant release, and cellular intake. In turn, starch—originating from plants—has shown promising results as a curcumin nanocarrier, improving its encapsulation efficacy and controlled release. Finally, cellulose can be used as a nanocarrier, due to its biocompatibility and hydrophilicity. In conducted studies, cellulose has proven its ability in the prolonged release of curcumin [91].

### 3.2. Curcumin-Targeted Exosomes

Exosomes are biological vesicles occurring in body fluids, in addition to being secreted by different mammalian cells in small amounts. Their endogenous function is, in general, the transport of different molecules that can be protected from the outside environment [101]. Therefore, they can be considered as biological NPs. By possessing low immunogenicity and high natural stability, they have a long half-life. In addition, they can cross the blood–brain barrier, making it easier to deliver drugs to the brain. Exosomes are more permeable in tumors and damaged tissues [102]. They are an alternative for both hydrophilic and lipophilic drug delivery. The problems associated with exosomes arise from their carrying capacity. In relation to curcumin, exosomes increase its solubility and bioavailability, enhancing its therapeutic efficacy. The protective capability of exosomes allows them to be the desirable curcumin nanocarriers. It has been proven that they can protect curcumin in gastrointestinal environment [91,103].

### 3.3. Curcumin-Loaded Lipid Nanoparticles

Lipid nanoparticles, such as solid lipid nanoparticles (SLN) and nanostructured lipid carriers (NLC), are useful nanoparticles to protect the payload against environmental conditions (e.g., pH variations, high temperature, enzymes), to provide the controlled release of the loaded drug and for targeted delivery [104,105]. SLN and NLC are biocompatible, biodegradable, and can be tailor-made for a series of biological targets, including cancer [16,106,107,108]. These particles comprise a range of selected solid lipids that have been screened for the production of curcumin-loaded SLN. The developed SLN were successfully tested against the growth of MCF-7 and BT-474 cell lines, in comparison to free curcumin solution [16]. SLNs, composed of a combination of trimiristine (Dynasan 114^®^), Propylene Glycol Caprylate (Sefsol-218^®^), and Pluronic F68^®^ loading 0.8% of curcumin, exhibited prolonged growth inhibition and cellular uptake in MCF-7 cells [109]. The oral bioavailability of curcumin was increased when loaded into PEGylated tristearin-SLNs [110]. The reported prolonged lipolysis of the SLNs was attributed to the interfacial hindrance promoted by the presence of polyethylene glycol chains into the surface of the particles (the so-called “PEGylation” approach). SLNs composed of stearic acid and lecithin, and loading curcumin at various concentrations (5, 10, 20, and 40 μM), showed a high cytotoxic effect against SKBR3 cells [111]. A high uptake of these particles by the cells was demonstrated in vitro. Curcumin-loaded SLNs enhanced apoptosis in the SKBR3 cells, in comparison with the free drug solution. The loaded particles promoted the ratio of Bax/Bcl-2, but decreased the expression of cyclin D1 and CDK4, opening a new perspective for the treatment of breast cancer.

Lipid nanoparticles can be applied in diverse medical fields [107,112]. They are more biocompatible than polymeric and inorganic nanocarriers [104,105,113]. Lipid nanoparticles can pass the blood–brain barrier [54,84]. This ability makes them crucial in the treatment of neurodegenerative diseases. Due to a flexible self-assembly process, they are conferred by a variety of morphological and structural characteristics [84]. There are two classical types of lipid-based nanoparticles, namely solid lipid nanoparticles (SLNs) and nanostructured lipid carriers (NLCs). NLCs differ from SLNs due to the nanostructured lipid matrix composed of a mixture of solid and liquid lipids (that melts above 40 °C), originating a less-ordered matrix with the capacity to load higher drug amounts than the SLNs, preventing their leakage during storage and allowing a more flexible drug release modulation. Both SLN and NLC formulations require surfactants (e.g., poloxamers, tweens) to stabilize the lipid matrices in aqueous dispersion [114]. A study was aimed at the evaluation of curcumin encapsulation by NLC and SLN. Based on pharmacokinetics, it has been shown that NLC were more efficient in both cases. In turn, another study, using rat brains and serum, has proven that the accumulation of curcumin was higher in NLC. A further investigation has evidenced that curcumin-loaded NLC is effective for brain cancer inhibition by increasing curcumin bioavailability [84,115].

On the other hand, lipid-based liquid crystalline nanoparticles (LCNPs) are formed through lipid self-assembly and have many promising properties as in vivo drug delivery carriers to heighten the bioavailability of both hydrophilic and hydrophobic drugs [116]. They exhibit thermodynamic stability. These nanocarriers are formed when lyotropic lipids with amphiphilic molecules are mixed together [117]. When LCNPs pass the blood–brain barrier, they exhibit enhanced bioavailability, reduced adverse effects, reduced degradation, and the controlled release of targeted drugs. A study on curcumin encapsulation into a monoolein-based LCNP has shown that the encapsulation process reached 100 wt.% efficiency, while curcumin colloidal stability was greatly enhanced [84].

### 3.4. Curcumin-Loaded Liposomes

Liposomes are another type of lipid carrier that has been shown to improve the stability, bioavailability, and targeted delivery of drugs. Liposomes are phospholipid bilayers, forming concentric vesicles that can also be tailor-made for site-specific targeting. A review by Feng et al. has discussed several approaches for the incorporation of curcumin in liposomes, using folic acid, retinoids, chitosan, cyclodextrins, and polyethylene glycol conjugates [118]. Combining curcumin with C6-ceramide in liposomes resulted in an enhanced antitumor effect against osteosarcoma cell lines [119]. Curcumin-loaded liposomes were coated with prostate-specific antigen antibodies and the obtained carriers could be used transcutaneously, as they are able to go through the hair follicles. Dimyristoyl phosphatidylcholine-based liposomes have shown around 70–80% inhibition in prostate cancer cell multiplication [120].

Liposomes consist of one or multiple lamellar lipid bilayers—usually phospholipids and aqueous cores—which allow them to carry lipophilic or hydrophilic compounds [54,91]. As nanocarriers, they are highly biocompatible and flexible. There is a risk of various liposomes fusing into one, therefore, a surface functionalization is used to avoid this process. In the bloodstream, liposomes may be covered by the protein corona [121,122]. Consequently, mononuclear phagocytic systems are activated, resulting in liposome removal. To counteract these phenomena, a surface functionalization enriched by polyethyleneglycol (PEG) reduces the amount of protein corona attached. If a liposome is cationic charged, the internalization of the liposome by the cell is improved because of the interaction with the negative charged cell membranes [91,123]. Recently, Tai et al. studied the stability and release performance of curcumin-loaded liposomes with varying contents of hydrogenated phospholipids [124].

### 3.5. Curcumin-Loaded PLGA Nanoparticles

Polymers consisting of different monomers that exhibit biocompatibility, high stability, small size, long plasma half-life, and effective carrying capacity are easily modifiable. Generally, they increase bioavailability, solubility, and therapeutic effect. Polymeric micelles can be used in several medical fields. Researchers have observed that curcumin has possessed both higher cytotoxic effect and prolonged release in a polymeric micelle [91]. For the treatment of neurological diseases, poly(lactic-co-glycolic acid) (PLGA), is the most employed copolymer. Curcumin-loaded PLGA nanoparticles have shown an increase in bioavailability in both oral and intravenous administrations [125]. An increase in curcumin in the brain over time, especially in the hippocampus and cerebral cortex, has shown a neuroprotective effect. PLGA nanocarriers can make it easier to cross the blood–brain barrier. Curcumin encapsulated in polymeric micelles are the hydrophobic cores. The micelles create an optimal environment for the transport of loaded drugs. Additionally, mixed polymeric micelles, enriched by curcumin, have better colloidal stability and higher loading capacity [84].

### 3.6. Curcumin-Cyclodextrin Complexes

Cyclodextrins (CDs) are macrocyclic host macromolecules with a hydrophobic interior. They can be used to transport hydrophobic molecules and a hydrophilic exterior. Due to their ability to extract lipids and proteins from cellular membranes, CDs can pass through the blood–brain barrier. It has been evidenced that these molecules can improve curcumin solubility [84]. Scientific research carried out by Yadav V.R. et al. (2009) has proven the anti-inflammatory activity of the cyclodextrin complex of curcumin for the treatment of inflammatory bowel disease (IBD) in a colitis-induced rat model. The study proved that the degree of colitis caused by the administration of dextran sulfate solution was significantly attenuated by the CDs of curcumin [126].

### 3.7. Other Curcumin-Containing Nanopharmaceuticals for Cancer

Cancer is the outcome of combined genetic and epigenetic changes induced by environmental/dietary factors [127] that cause the inappropriate activation or inactivation of specific genes, leading to neoplastic transformation [128]. These consequences consist of a lineage of genetically and/or phenotypically different cell types (cell heterogeneity) that arise over a period of time [54], followed by apoptosis, uncontrolled cell proliferation, metastasis, and angiogenesis [127]. In order to create efficient anticancer therapy, plenty of intensive research has been conducted [54]. Currently, there are commonly used methods, such as radiotherapy, immunotherapy, chemotherapy [91], surgery, and hormonal therapy [127]. The most frequently used chemotherapy is effective in most of the treatments, however it induces side effects and leads to intrinsic and acquired drug resistance, which is magnified by the high degree of molecular heterogeneity of tumors [127]. Fundamental factors, such as environmental, dietary, occupational, and epigenetic factors, influence the carcinogenic process, as they can determine a period of latency.

An alternative strategy to control the development of cancer can be the intervention with phytochemicals that have little or no toxicity [54]—similar to curcumin. As a promising agent that can promote cells protection with apoptotic potential towards several types of cancer cells, curcumin can be used both in cancer prevention and therapy [91,129,130,131]. Nanotechnology has acquired an important role in the development of advanced drug delivery carriers as a way of providing the targeted drug delivery of curcumin that can accompany chemotherapeutic treatment [127]. Therefore, this bioactive component has become a promising additional therapeutic option for cancer patients [54]. Nanotechnology application within cancer research may bring numerous advantages, as well as improve cancer therapy and detection, earlier biomarker identification, and advanced knowledge of the mechanism of tumor progression [132]. Nanostrategies can be designed and personalized to the selected cancer treatment by improved drug delivery, reduced toxicity, enhanced safety profile, targeted therapy, and extended product life-cycles [127].

The major complexity of cancer physiology highly reduces the efficacy of monotherapy strategy through the acquisition of drug resistance and possible tumor recurrence. Due to those mentioned above, a combination of chemotherapy would be the easiest approach to overcome this issue, which concerns non-overlapping toxicity, non-cross resistance, and enhanced tumor cell killing efficacy. It is considered that nanocarriers are able to help further overcome mono-therapeutic complications [127,132].

The multidrug resistance (MDR) of cancer cells includes a modified apoptosis pathway and an overexpression of multidrug transporters, such as p-glycoprotein (P-gp), which comprises one of the most important problems to overcome in cancer treatment. The co-delivery of chemo-sensitizing and chemotherapeutic agents is a good approach to disrupt MDR and to overcome the negative impact of anticancer drugs. In order to overcome MDR, a vast number of nanoparticle formulations are designed to include a combination of drugs with chemo-sensitizers, MDR cytotoxic, and modulating agents [127].

Over the last few years, the basis of combination drug therapy consisted of nanoparticles with multiple chemotherapeutic agents and their inherent mechanisms of action. This approach helped to reduce side effects and to permit specific interactions with a target site for a different type of cancer [133]. Formerly, almost all of the complex nanoparticles contained water-soluble drugs. Nowadays, the development of a sophisticated nanoparticle-based drug delivery system made it possible to deliver both hydrophobic and hydrophilic drugs incorporated in one nanosystem [127].

Combination therapy can induce a different effect when compared to the use of individual drugs, but can also induce antagonism in complex interactions. The design of a nanoparticle combination needs to take count of all these effects to acquire a variety of favorable outcomes, such as the reduction in side effects, drug-resistance prevention, a decrease in the dose of individual drugs, and enhancing efficacy [127].

Curcumin has common action on several modulators inducing anti-cancer effects. Both alone or in combination with other drugs, it is nontoxic and aimed to enhance the therapeutic efficiency of chemotherapeutics by inhibiting ATP-binding cassette (ABC) efflux transporters [134]. Curcumin inhibits tumor growth by arresting cell cycle progression. It also induces apoptosis, inhibits the expression of anti-apoptotic proteins, and inhibits multiple cell survival signaling pathways and their cross-communication, as well as modulating immune responses. All these characteristics make it a desired drug for the mono- or combination therapy of several kinds of cancer (e.g., osteosarcoma, leukemia, gastrointestinal cancer, prostate or cervical cancers, and lung cancers [127,132]).

Curcumin has also shown activity in the methylation process of DNA with the so-called DNA methyltransferases (DNMT). Studies developed by Maugeri et al. suggest that curcumin restores ROS production, as well as DNMT functions, which is interesting for the treatment of several diseases caused by a high concentration of oxidative stress [135]. To develop an effective anticancer combination therapy, the investigation into the inherent obstacles is mandatory. Based on the efficacy and pharmacokinetic profile of each drug, initial studies require an exact harmonization of the optimal mass ratio of each drug in a combination, making it hard to develop a curcumin anticancer combination therapy [134]. Another important obstacle is the non-site-specific combination nanoparticulate formulation that has been recently fixed by the combination of folate or other specific antigens as a way of improving site specificity for the delivery of cytotoxic drugs to tumor cells. Likewise, the short- and long-term toxicity should be tested as some components used for fabricating nanoformulations are toxic. The quality of the nanocarrier-based combination therapies should also be evaluated by design parameters, rationally based on the size and surface physicochemical properties of the materials to be fabricated [15,127].

The use of microemulsions, based on different oil compositions, have also been proposed for the loading of curcumin. Microemulsions are thermodynamically stable and transparent systems, having a surfactant/oil ratio ≥2 and small diameter (≤30 nm) [136]. The solubility and bioavailability of curcumin were improved when loaded into microemulsions consisting of Capryol 90 (oil), Cremophor RH40 (surfactant), and Transcutol P aqueous solution (co-surfactant) [137]. Calligaris et al., tested different lipids (sunflower, peanut, castor, and extra virgin olive oil, tristearin, and tripalmitin) to produce curcumin-loaded microemulsions [138]. The authors concluded that the oil type influenced the curcumin degradation rate, which was lower in the tristearin-containing microemulsions than in those containing extra virgin olive oil. This result was attributed to the protective effect promoted by the presence of solid lipid in the inner lipid phase of the microemulsions. A soybean oil microemulsion containing 3 mg/mL curcuminoid extract was prepared and was shown to strongly inhibit the growth of colon cancer cells HT-29 through the elevation of p53 expression and p21-independent route, the inhibition of both cyclin A and CDK2 expressions for cell cycle arrest at S phase, and the increase in cytochrome C expression, as well as the activities of caspase-8, caspase-9, and caspase-3 [139]. These results open the perspective of using microemulsions containing curcumin for the treatment of colon cancer.

Eudragit RS 100 nanoparticles were loaded with quantum dots (QDs) and curcumin and their effect on cancerous and bacterial cells were compared to curcumin nanoparticles only [140]. The authors have confirmed that the combined loading of QDs and curcumin significantly inhibited the growth of both breast (MCF-7) and colon (HCT-116) tumor cell lines, in comparison to free and single loaded curcumin. Moreover, the nanoparticle treatment of normal cells (HEK-293) was not affected. These results open the perspective for the site-specific targeting of curcumin nanoparticles for chemotherapy.

Dextran–curcumin conjugate nanoparticles were recently prepared via enzyme chemistry with immobilized laccase, acting as a solid biocatalyst for the combined loading of methotrexate and curcumin [141]. The developed nanoparticles were able to internalize MCF-7 cells effectively, attributed to the synergistic effect of curcumin and the chemotherapeutic compound.

Studies have shown that drug release can be enhanced through external energy sources, such as ultrasonic waves. Implantable drug delivery systems slowly release the drug, while prolonging the release time to last days, months, or even years [142]. There are two types of implantable drug delivery systems, namely the matrix type and the reservoir type. The matrix type implant is regarded as the safest system that withholds the drug uniformly scattered throughout the implant and is released through the erosion of the matrix with two phases. The erosion of the first phase had the objective of rapidly reaching the therapeutic concentration, and of the second phase, releasing the drug slowly to maintain that therapeutic concentration. The reservoir type implant—in which the rate of drug release is controlled by a semi-permeable polymeric membrane— is dependent on water influx. These kinds of implants are not recommended due to a high risk of dose dumping. In a study, a matrix type implant was prepared with curcumin and other phytochemicals. There was a biphasic curcumin release, followed by a slow controlled release. This has allowed for the achievement of high local curcumin concentration and systemic diffusion [54].

## 4. Conclusions

Curcumin, naturally occurring in the rhizome of turmeric, has multiple therapeutic effects in advanced diseases, mainly in the treatment of cancer, cancer prevention, neurodegenerative diseases, and gastrointestinal diseases. Despite many advantages, curcumin has various limitations, including low bioavailability and water solubility, which cause many difficulties during absorption. Due to these limitations, an alternative way of curcumin administration has been investigated in order to make it possible to use it as a therapeutic drug. Thereupon, different nanoparticles have attracted much interest as potential effective carriers for this bioactive component. Several types of nanoparticles loaded with curcumin were developed and studied to find the most suitable nanosystem for treating and preventing certain types of cancer and other diseases. It has been proven that curcumin can enhance the therapeutic efficiency of chemotherapeutics by inhibiting the ABC efflux transporter, and hence, helping with the treatment of cancer. Besides, after determining the right dosage, the anticancer activity of combination nanoparticles must be tested in cancerous cells and conditions as a way of evaluating treatment efficacy. This efficacy is often determined by the regression of angiogenesis, which is a mechanism of survival of cancerous cells. In neurodegenerative diseases (such as Alzheimer’s or Parkinson’s), some curcumin-loaded nanoparticles can cross the blood–brain barrier and help in the reduction in the inherent side effects or even reduce the β-amyloid plaque. Despite all of curcumin’s promising properties, there is still a need for more research.
